# Polyunsaturated Fatty Acids and Risk of Ischemic Stroke

**DOI:** 10.3390/nu11071467

**Published:** 2019-06-27

**Authors:** Stine Krogh Venø, Erik Berg Schmidt, Christian Sørensen Bork

**Affiliations:** 1 Department of Cardiology, Aalborg University Hospital, 9000 Aalborg, Denmark; 2 Department of Clinical Medicine, Aalborg University, 9000 Aalborg, Denmark

**Keywords:** polyunsaturated fatty acids, ischemic stroke, observational prospective studies, biomarkers

## Abstract

Ischemic stroke is a major cause of death and morbidity worldwide. It has been suggested that polyunsaturated fatty acids (PUFAs) may be associated with a lower risk ischemic stroke, but this has been far less studied than their role for coronary heart disease. In this paper, we summarize the main findings from previous follow-up studies investigating associations between intake or biomarkers of the major PUFAs including alpha-linolenic acid (ALA), marine n-3 PUFAs and linoleic acid (LA) and the development of ischemic stroke. Several follow-up studies have suggested that marine n-3 PUFAs may be associated with a lower risk of ischemic stroke although results have not been consistent and limited knowledge exist on the individual marine n-3 PUFAs and ischemic stroke and its subtypes. The role of ALA is less clear, but most studies have not supported that ALA is appreciably associated with ischemic stroke risk. Some studies have supported that LA might be associated with a lower risk of total ischemic stroke, while limited evidence exist on PUFAs and ischemic stroke subtypes. The associations may depend on the macronutrients that PUFAs replace and this substitution aspect together with focus on dietary patterns represent interesting areas for future research.

## 1. Introduction

Ischemic stroke is characterized by an episode of neurological dysfunction attributed to cerebral infarction in the perfusion territory of an stenosed or occluded artery [1]. Ischemic stroke is a major cause of death and disability worldwide, but some studies have indicated that adherence to a healthy diet and lifestyle may be of importance for reduction of ischemic stroke risk [2,3].

An important part of a healthy diet is believed to be an increased intake of polyunsaturated fatty acids (PUFAs). PUFAs are organic acids that naturally contains two or more double bonds in their carbon chain and are named according to the number, configuration and position of these double bonds [4]. PUFAs are upon ingestion incorporated into cell membranes and storage pools, utilised in energy production or converted into longer and more unsaturated fatty acids, which may give rise to biological compounds that may affect a variety of biological processes [5]. PUFAs have traditionally been divided into n-3 PUFAs and n-6 PUFAs and these two families each consist of fatty acids with varying carbon chain length and degree of unsaturation, which are important for their physiological and biological properties [4]. The major n-3 PUFAs include the short-chain plant-derived n-3 PUFA, alpha-linolenic acid (ALA, 18:3n-3), and the longer-chain marine n-3 PUFAs eicosapentaenoic acid (EPA, 20:5n-3), docosapentaenoic acid (DPA, 22:5n-3) and docosahexaenoic acid (DHA, 22:6n-3).

The major dietary n-6 PUFA is linoleic acid (LA, 18:2n-6) and together with ALA are the PUFAs consumed in largest amounts. The typical intake of LA in Western populations range between 10 to 17 g/d, while the average intake of ALA varies between 0.5 to 2.3 g/d [5,6,7]. The major sources of LA include plant oils, meat and eggs [6], while ALA is found in large amounts in some plant oils, but can be also be found in walnuts, green vegetables, whole-grain cereals, margarines, dairy products and meat [8,9,10]. LA and ALA are precursors for further desaturation and elongation products along a shared metabolic pathway. LA can thus be converted into arachidonic acid (AA, 20:4n-6), while ALA can be converted into LC n-3 PUFAs, however, the conversion efficiency seems to be limited in humans and influenced by gender, genetics and intake of other fatty acids [5,11]. The LC n-3 PUFAs can be acquired from seafood and especially fatty fish and their biological effects has been extensively studied over the recent decades since Dyerberg and colleagues hypothesized that LC n-3 PUFAs in particular EPA were protective against atherothrombosis [12,13,14].

LC n-3 PUFAs has been ascribed several effects that might be beneficial in the prevention of ischemic stroke including lowering of triglycerides, inflammation, blood pressure and platelet aggregability, and improved endothelial function and atherosclerotic plaque stability, which likely are mediated by a combination of mechanisms [4,14,15,16,17]. On the other hand the biological effects of other PUFAs have been less studied, but few studies have suggested that ALA might be associated with anti-inflammatory and anti-atherosclerotic properties [18]. Whether these suggested effects is mediated by ALA per se or can be attributed to its role as a precursor for LC n-3 PUFAs, however, remains unclear. LA has been reported to be associated with lowering of low-density lipoprotein (LDL) cholesterol, blood pressure and insulin resistance [19], all important for atherosclerotic risk. Concerns have, however, been raised that LA could confer a higher risk of vascular disease due to its role as a precursor of AA that gives rise to eicosanoids considered pro-inflammatory and pro-thrombotic including formation of proinflammatory leukotriene B4 from leukocytes and proaggregatory and vasoconstrictive thromboxane A2 [4]. However, stable isotope studies and clinical trials have indicated that high intakes of LA do not seem to significantly increase tissue levels of AA or increased production of inflammatory mediators [20]. Indeed, there is actually some indications that LA might possess anti-inflammatory effects [20,21].

Several studies have suggested that LC n-3 PUFAs and LA may be associated with a lower risk of coronary heart disease (CHD) [4,22,23,24]. Although, CHD and ischemic stroke may share pathophysiological similarities, less is known about the role of PUFAs in relation to risk of ischemic stroke and previous studies have not yielded consistent results.

The objective of this paper was to summarize the main findings from previous follow-up studies investigating associations between intake or biomarkers of exposure of the major PUFAs (ALA, LC n-3 PUFAs, and LA) and ischemic stroke.

## 2. ALA and Ischemic Stroke

Few studies have investigated the association between ALA intake and ischemic stroke [25,26,27,28,29] (Table 1). In the Monitoring Project on Risk Factors for Chronic Diseases Study (MORGEN Study), dietary intake of ALA was inversely associated with the rate of total ischemic stroke among Dutch men and women [25]. In the Cardiovascular Health Study (CHS), indications of a lower rate of total ischemic stroke were observed when comparing the highest quintile of ALA intake with the lowest, but no consistent pattern of association was observed across quintiles of ALA in this cohort of US men and women [26]. Further, no clear association was observed between ALA intake and the rate of total ischemic stroke among women enrolled into the Women’s Health Study [27] or the Swedish Mammography Cohort [28] and among men and women enrolled into the Diet, Cancer and Health (DCH) cohort [29]. Limited knowledge is available regarding the associations between ALA intake and the risk of ischemic stroke subtypes but no appreciable pattern of associations were observed between intake of ALA and the rate of ischemic stroke subtypes caused by large artery atherosclerosis, small vessel-occlusion or cardioembolism in the DCH cohort [29] (Table 2).

The findings from previous follow-up and nested-case control studies using biomarkers of ALA to investigate associations with ischemic stroke have not been conclusive [26,31,32,33,38,39,40] (Table 3). In a large case-cohort study based on data from the DCH cohort we reported indications of a U-shaped pattern of association between adipose tissue content of ALA and the rate of total ischemic stroke although not statistically significant [33]. A follow-up study based on the Finish Kuopio Ischaemic Heart Disease Risk Factors (KIHD) study also reported indications of a U-shaped pattern of association between quartiles of ALA content in serum and total ischemic stroke, while a nested case-control study reported a modest non-significant inverse association between ALA in serum and total ischemic stroke [32,40]. In contrast, several other biomarker studies did not find ALA content in serum, plasma phospholipids or cholesterol esters associated with the risk of total ischemic stroke [26,31,38,39].

Limited data are available on biomarkers of ALA and ischemic stroke subtypes (Table 2). However, data from the DCH cohort suggested a statistically significant U-shaped association between ALA content in adipose tissue and the rate of ischemic stroke due to large artery atherosclerosis with the lowest rate observed around the median content of ALA in adipose tissue [33]. In contrast, a positive statistically non-significant association was observed between ALA content in adipose tissue and the rate of ischemic stroke due to cardioembolism, whereas no appreciable association was found between ALA in adipose tissue and ischemic stroke due to small-vessel occlusion [33].

In summary, previous studies evaluating either intake of ALA or content of ALA in blood components or adipose tissue have shown conflicting results, but most studies does not support that ALA exposure is inversely associated with ischemic stroke risk.

## 3. Marine LC n-3 PUFAs and Ischemic Stroke

Several studies have investigated the association between intake of LC n-3 PUFAs and total ischemic stroke [27,28,30,35,41,42,43,44,45,46] (Table 4) and has recently been reviewed [47]. In brief, findings from the Nurses’ Health Study (NHS) indicated a statistically non-significant inverse pattern of associations between EPA+DHA intake and the rate of total ischemic stroke [30]. Analyses of ischemic stroke subtypes showed an inverse pattern of associations between EPA+DHA intake and lacunar infarctions, while no clear associations were observed for large artery occlusive infarctions [30] (Table 2). Lower rates of total ischemic stroke across quintiles of EPA+DHA intake were observed in the Swedish Mammography cohort [28] and in the Health Professionals Follow Study (HPFS) although results were not statistically significant in the latter when comparing the highest quintile of intake with the lowest [41]. In the MORGEN study, gender-specific analyses indicated lower rates of total ischemic stroke in subjects with a high EPA+DHA intake, but the confidence intervals were wide and the point estimates were not statistically significant [42]. A recent study from our groups by Venø et al. [35] investigated associations between dietary intake of total marine LC n-3 PUFAs and of individual marine n-3 PUFAs and the rate of total ischemic stroke and ischemic stroke subtypes using data from the DCH cohort. In dietary analyses, no appreciable associations were observed between intake of total and individual n-3 PUFAs, EPA or DHA and the rate of total ischemic stroke [35]. Also, in five other follow-up studies, no consistent pattern of associations between EPA+DHA intake and rate of total ischemic strokes were reported [27,43,44,45,46]. However, in the study by Venø et al. a high intake of total n-3 PUFAs, EPA and DHA was statistically significantly associated with lower rates of ischemic stroke due to large artery atherosclerosis when comparing the highest quartile of intake with the lowest [35] (Table 2). In contrast, indications of higher rates of ischemic stroke due to cardioembolism were observed with intake of LC n-3 PUFAs, whereas no consistent pattern of association was observed between LC n-3 PUFAs and the rate of ischemic stroke due small-vessel occlusions [35] (Table 2).

Some biomarker studies have investigated the associations between levels of LC n-3 PUFAs in blood compartments or adipose tissue and the risk of ischemic stroke [31,32,34,35,38,39,40,48] (Table 5). Thus, a the content of EPA, DPA and DHA in plasma phospholipids was analysed in relation to ischemic stroke and its subtypes using pooled data from the CHS, NHS and HPFS [34]. Lower rates of total ischemic stroke was observed when comparing the highest quintile of content of DPA and DHA with the lowest, respectively, while no appreciable association was found for EPA [34]. However, the associations seemed to depend on the ischemic stroke subtype in question (Table 2). In analyses of ischemic stroke subtypes, DHA was associated with the rate of atherothrombotic strokes (large artery and small-vessel infarctions), whereas no association was found for EPA and DPA. However, DPA was associated with lower rates of cardioembolic strokes, whereas no associations were observed between EPA or DHA and the rate of cardioembolic stroke [34]. Few other studies have supported that circulating biomarkers of LC n-3 PUFAs might be associated with a lower risk of ischemic stroke although results have not been consistent [31,32,38,40,48] (Table 5).

The associations between adipose tissue content of LC n-3 PUFAs and the risk of ischemic stroke and ischemic stroke subtypes were investigated based on data from the DCH cohort and no appreciable association was observed between total adipose tissue of LC n-3 PUFAs and the rate of total ischemic stroke [35]. However, the EPA content in adipose tissue was inversely associated with the rate of total ischemic stroke when comparing the highest with the lowest quintile [35]. In contrast, adipose tissue content of DPA seemed to be associated with a higher rate of total ischemic stroke, whereas no association was found for DHA [35]. In analyses of ischemic stroke subtypes, the content of EPA in adipose tissue was inversely associated with the rate of ischemic stroke due to large artery atherosclerosis and small-vessel occlusions, while a higher rate of ischemic stroke due to cardioembolism was noted when comparing the highest quartile with the lowest although not statistically significant [35] (Table 2). Adipose tissue content of DPA and DHA was associated with higher rates of ischemic stroke caused by cardioembolism whereas no consistent pattern of association was found for ischemic stroke caused by large artery atherosclerosis or small-vessel occlusions [35] (Table 2).

In summary, several large prospective studies evaluating EPA and/or DHA intake or their content in blood components or adipose tissue have suggested an inverse association although results have not been consistent. The associations between LC n-3 PUFAs and ischemic stroke subtypes may differ with the most beneficial results observed in subjects with strokes of atherosclerotic etiology.

## 4. LA and Ischemic Stroke

Studies of the association between dietary intake of LA and the rate of ischemic stroke are sparse (Table 6). In analyses based on data from the Swedish Mammography Cohort, no association was observed between intake of LA+AA and the rate of total ischemic stroke [28]. Also, no clear association was observed between LA+AA intake and the rate of total ischemic stroke in another Swedish cohort [45]. However, the intake of LA and AA was not analysed separately and the macronutrient(s) to be replaced for n-6 PUFAs was not specified in these studies. In contrast to n-3 PUFAs, LA constitute a significant proportion of total energy intake and a higher intake of LA must necessarily be accompanied by a lower intake of other macronutrients in an isocaloric setting. In a follow-up study using statistical substitution models we therefore investigated the risk of total ischemic stroke and ischemic stroke subtypes with a 5% higher intake of LA and a concomitant lower intake from saturated fatty acids (SFAs), monounsaturated fatty acids (MUFAs) or glycemic carbohydrates using data from the DCH cohort [36]. This study suggested that replacing MUFAs or glycemic carbohydrates with LA might be associated with a lower risk of total ischemic stroke although not statistically significantly [36]. Further, in analyses of ischemic stroke subtypes, replacement of SFA with LA indicated a lower rate of ischemic stroke due to large artery atherosclerosis and replacement of glycemic carbohydrates with LA indicated a lower rate of ischemic stroke due to small-vessel occlusions, although the observed associations were not statistically significant [36] (Table 2). A statistically significant lower rate of ischemic stroke due to small-vessel occlusions was observed when LA replaced MUFAs [36] (Table 2).

Several prospective biomarker studies have supported that circulating or adipose tissue content of LA may be associated with a lower risk of ischemic stroke although results have not been consistent [23,31,32,37,38,39,40,49] (Table 7). A previous study by Iso et al. suggested a lower odds of total ischemic stroke and lacunar infarctions per 1 SD increase in LA content in serum [31]. Also, in analyses based on data from the ARIC Study, a statistically significant inverse association between LA content in plasma cholesterol esters and the rate of total ischemic stroke and indications of an inverse association was observed between LA content in plasma phospholipids and the rate of total ischemic stroke [39]. Also, several other studies have indicated a lower risk of total ischemic stroke with the content of LA in serum, plasma phospholipids and cholesterol esters, but these findings were not statistically significant and not entirely consistent [32,38,40,49]. However, a large case-cohort study based on data from the DCH study found a dose-dependent inverse association between adipose tissue content of LA and the rate of total ischemic stroke [37]. In analyses of ischemic stroke subtypes, adipose tissue content of LA was inversely associated with the rate of ischemic stroke due to large artery atherosclerosis and indications of lower rates of ischemic stroke due to small-vessel occlusions was observed, whereas no clear association was found between LA in adipose and the rate of ischemic stroke due to cardioembolism [37]. Recently, a harmonized individual-level analysis based on prospective data from the Fatty Acid and Outcome Research Consortium (FORCE) found an overall lower rate of total ischemic stroke with higher levels of LA in blood compartments and adipose tissue in analyses including 3705 incident ischemic stroke cases [23].

In summary, limited evidence regarding LA intake and ischemic stroke exist, but LA intake might be associated with a lower risk of total ischemic stroke although the association may depend on macronutrient used to replace LA and the ischemic stroke subtype in question. Several biomarker studies have supported that a high content of LA in blood components or adipose tissue may be associated with a lower risk of total ischemic stroke.

## 5. Discussion

Several prospective follow-up studies have suggested that LC n-3 PUFAs perhaps in particular EPA and DHA may be associated with a lower risk of total ischemic stroke although results have not been consistent. A few studies have suggested that the associations between individual LC n-3 PUFAs may differ amongst subtypes of ischemic stroke with the most beneficial findings observed in ischemic strokes of presumed atherosclerotic origin. Regarding the role of ALA for development of ischemic strokes, most prospective studies have not supported that ALA may be appreciably associated with a lower risk of ischemic stroke. The associations between LA and ischemic stroke have been less studied than the role of n-3 PUFAs, but some biomarker studies have supported that LA might be associated with a lower risk of ischemic stroke. Limited evidence exist regarding LA intake and ischemic stroke, but the association may depend on the macronutrient used to replace LA.

Biomarkers of PUFA exposure may represent useful complementary measures of exposure to investigate the associations with chronic diseases such as ischemic stroke [50]. Dietary studies using estimated PUFA intakes as exposure are limited by measurement error, which may result in loss of statistical power and attenuation of associations toward null. In contrast, the content of PUFAs in blood components or adipose tissue can be assessed more precise and are considered objective biomarkers of exposure [50,51]. Long-term measures of exposure is likely to be of greater etiological relevance in the development of ischemic stroke compared to short-term measures of exposure. Adipose tissue is considered the gold standard due to a slow turnover time possibly reflecting intake of PUFAs during the previous 1–2 years, whereas shorter-term biomarkers such as PUFA content in blood components may represent the exposure up to few months. However, the content of PUFAs in blood components or adipose tissue reflects both intake and metabolism [50]. Also, direct comparison of measures of associations obtained in previous biomarker of PUFAs from different population should be done with caution because differences in PUFA content in human tissue may be largely influenced by differences in the underlying dietary pattern [50,51].

Finally, ischemic stroke is a heterogeneous condition and it is likely that the biological effects of individual LC n-3 PUFAs and LA may differ across subtypes of ischemic stroke of different etiology. Ischemic stroke due to large artery atherosclerosis is considered to be of atherosclerotic origin, whereas ischemic stroke due to small-vessel occlusion may develop as a result of atherosclerosis or lipohyalinosis affecting the smaller penetrating arteries in the brain [52,53]. In contrast, ischemic cardio-embolic strokes are mainly caused by emboli arising from the heart due to arrhythmias, particularly atrial fibrillation or flutter. Furthermore, ischemic stroke may in rare cases develop as a result of nonatherosclerotic vasculopathies or prothrombotic disorders. Thus, these rather distinct potential causes of ischemic stroke underline that separate analyses of ischemic stroke subtypes may contribute with a better understanding of the underlying biology of exposure to PUFAs. Recent results based on data from the DCH cohort [35] and the pooled analyses by Saber et al. [34] indicated that the associations between individual LC n-3 PUFAs and ischemic stroke subtypes may differ. Interestingly, results from the DCH cohort indicated stronger associations for EPA and LA on ischemic stroke due to large artery atherosclerosis than for ischemic stroke due to small-vessel occlusion. This might indicate that potential anti-atherosclerotic properties of marine n-3 PUFAs may be of importance. Anti-atherosclerotic properties of LC n-3 PUFAs may include lowering of triglycerides and perhaps also of atherogenic small-dense LDL particles, a reduction in inflammation and blood pressure, while LA might inhibit the atherosclerotic process by lowering of LDL-cholesterol and blood pressure. EPA seemed to be stronger associated ischemic stroke due to large artery atherosclerosis than DHA in the DCH cohort, but whether these differences can be attributed to different biological effects remain unclear, but is not unlikely as many data support that a major effect of DHA on heart disease may be an antiarrhythmic effect.

In addition, the associations may depend on the macronutrients used to replace PUFAs and such substitution aspects together—with focus on dietary patterns—represent interesting areas for future research.

## Figures and Tables

**Table 1:** Characteristics of observational follow-up studies investigating intake of alpha-linolenic acid (ALA) and total ischemic stroke.

Author and Study	Study Population and Country	Sex	No. of Participants	Baseline Age (yrs)	Exposure Assessment	Intake (g/d)	No. of Cases	Follow-up (yrs)	Hazard Ratios (95% CI)	Variables Controlled for
De Goede et al. 2011 [25]	The MORGEN Study cohort; The Netherlands	M+W	19,896	20–65	FFQ	Q1: 1.0 Q3: 1.3 Q5: 1.9	144	10.5	Q2: 0.63 (0.39; 1.02) Q3: 0.45 (0.26; 0.77) Q4: 0.55 (0.33; 0.92) Q5: 0.70 (0.43; 1.12)	Age; sex; BMI; total energy intake; cigarette smoking; educational level; parental history of MI; alcohol.
Larsson et al. 2012 [28]	The Swedish Mammography Cohort; Sweden	W	34,670	49–83	FFQ	Q1: 0.9 Q3: 1.1 Q5: 1.5	1310	10.4	Q2: 0.99 (0.83; 1.17) Q3: 1.03 (0.86; 1.22) Q4: 1.00 (0.84; 1.19) Q5: 1.11 (0.93; 1.32)	Age; smoking status and pack-years of smoking; education; BMI; physical activity; hypertension; diabetes; aspirin; family history of MI; intakes of alcohol, protein, fiber and cholesterol.
Fretts et al. 2014 [26]	The CHS cohort; United States	M+W	2583	≥65	FFQ	Q1: 1.3% ^1^ Q3: 1.8% ^1^ Q5: 2.4% ^1^	278	9.6 ^2^	Q2: 0.89 (0.61; 1.30) Q3: 0.84 (0.58; 1.22) Q4: 1.08 (0.75; 1.54) Q5: 0.70 (0.47; 1.04)	Age; sex; total energy intake; race; enrollment site; education; smoking status; diabetes; BMI; waist circumference; physical activity; alcohol; treated hypertension.
Rhee et al. 2017 [27]	The WHS cohort; United States	W	38,392	≥45	FFQ	Q1: 0.7% ^3^ Q3: 1.0% ^3^ Q5: 1.4% ^3^	807	18.2 ^2^	Q2: 0.89 (0.71; 1.13) Q3: 0.89 (0.71; 1.12) Q4: 1.05 (0.84; 1.30) Q5: 1.02 (0.82; 1.27)	Age; randomized treatment assignment; BMI; smoking; alcohol; physical activity; oral contraceptive use; HRT; multivitamins; total energy intake; family history of MI; hypertension; high cholesterol; diabetes.
Bork et al. 2018 [29]	The DCH cohort; Denmark	M+W	55,018	50–65	FFQ	Q1: 1.2 Q3: 1.8 Q5: 2.6	1859	13.5	Q2: 1.03 (0.87; 1.21) Q3: 1.12 (0.95; 1.31) Q4: 1.03 (0.87; 1.22) Q5: 1.10 (0.93; 1.31)	Age; gender; length of schooling; smoking; physical activity; waist circumference adjusted for BMI; alcohol.

Abbreviations: MORGEN, The Monitoring Project on Risk Factors for Chronic Diseases Study; M, men; W, Women; FFQ, Food Frequency Questionnaire; Q, Quintile; CI, Confidence interval; BMI, Body mass index; MI, Myocardial infarction; CHS, Cardiovascular Health Study; WHS, Women’s Health Study; HRT, Hormone replacement therapy; DCH, Diet, Cancer and Health. The presented measures of association was selected for models that included adjustment for lifestyle factors without including dietary covariates whenever possible. The lowest quintile of intake was used as reference. ^1^ Percentage of total fatty acids; ^2^ Follow-up time was calculated as person-years of follow-up divided by the total number of participants; ^3^ Percentage of total energy intake.

**Table 2:** Measures of association obtained in observational follow-up and nested case-control studies investigating intake or biomarkers of major PUFAs in relation to ischemic stroke subtypes.

Author and Exposures of Interest	Large Artery Occlusive Infarctions	Small-Vessel and Lacunar Infarctions	Cardio-Embolic Infarctions
Iso et al. 2001 [30]			
No. cases	88	142	
EPA+DHA intake	HR Q2-5 vs. Q1: Q2: 0.56 (0.28; 1.10) Q3: 0.50 (0.25; 1.02) Q4: 0.96 (0.51; 1.81) Q5: 1.09 (0.52; 2.29)	HR Q2-5 vs. Q1: Q2: 0.89 (0.55; 1.43) Q3: 0.59 (0.34; 1.00) Q4: 0.67 (0.39; 1.14) Q5: 0.37 (0.19; 0.73)	
Iso et al. 2002 [31]			
No. of cases	19	95	8
ALA in serum	OR: 0.82 (NS, CI not re-ported) per 1 SD increase ^1^	OR: 0.82 (NS, CI not re-ported) per 1 SD increase ^1^	OR: 0.89 (NS, CI not re-ported) per 1 SD increase ^1^
EPA in serum	OR: 2.28 (1.14; 4.57) per 1 SD increase ^1^	OR: 0.89 (NS, CI not re-ported) per 1 SD increase ^1^	OR: 0.63 (NS, CI not re-ported) per 1 SD increase ^1^
DPA in serum	OR: 1.35 (NS, CI not re-ported) per 1 SD increase ^1^	OR: 1.17 (NS, CI not re-ported) per 1 SD increase ^1^	OR: 1.59 (NS, CI not re-ported) per 1 SD increase ^1^
DHA in serum	OR: 1.53 (NS, CI not re-ported) per 1 SD increase ^1^	OR: 1.02 (NS, CI not re-ported) per 1 SD increase ^1^	OR: 1.06 (NS, CI not re-ported) per 1 SD increase ^1^
LA in serum	OR: 0.66 (NS, CI not re-ported) per 1 SD increase ^1^	OR: 0.63 (0.46; 0.88) per 1 SD increase	OR: 0.92 (NS, CI not re-ported) per 1 SD increase ^1^
Yaemsiri et al. 2013 [32]			
No. of cases	96	250	209
EPA in serum	OR: 0.51 (0.23; 1.14) per 1 SD increase ^2^	OR: 0.58 (0.36; 0.94) per 1 SD increase ^2^	OR: 0.84 (0.55; 1.30) per 1 SD increase ^2^
DPA in serum	OR: 0.83 (0.47; 1.47) per 1 SD increase ^2^	OR: 0.64 (0.44; 0.94) per 1 SD increase ^2^	OR: 0.82 (0.55; 1.24) per 1 SD increase ^2^
DHA in serum	OR: 0.70 (0.36; 1.36) per 1 SD increase ^2^	OR: 0.61 (0.40; 0.93) per 1 SD increase ^2^	OR: 0.66 (0.43; 1.02) per 1 SD increase ^2^
Bork et al. 2018 [29]			
No. of cases	316	835	102
ALA intake	HR Q2-5 vs. Q1: Q2: 1.03 (0.71; 1.51) Q3: 0.91 (0.62; 1.34) Q4: 0.90 (0.61; 1.34) Q5: 0.96 (0.65; 1.44)	HR Q2-5 vs. Q1: Q2: 1.08 (0.84; 1.39) Q3: 1.23 (0.97; 1.56) Q4: 1.09 (0.84; 1.40) Q5: 1.25 (0.98; 1.61)	HR Q2-5 vs. Q1: Q2: 1.30 (0.62; 2.72) Q3: 1.23 (0.59; 2.56) Q4: 1.08 (0.51; 2.29) Q5: 1.31 (0.63; 2.76)
Bork et al. 2018 [33]			
No. of cases	297	772	99
ALA in adipose tissue	HR Q2-5 vs. Q1: Q2: 0.72 (0.48; 1.08) Q3: 0.63 (0.41; 0.96) Q4: 0.83 (0.56; 1.22) Q5: 0.95 (0.65; 1.40)	HR Q2-5 vs. Q1: Q2: 1.00 (0.77; 1.31) Q3: 0.96 (0.73; 1.25) Q4: 1.02 (0.78; 1.33) Q5: 1.05 (0.80; 1.38)	HR Q2-5 vs. Q1: Q2: 1.05 (0.53; 2.06) Q3: 1.16 (0.59; 2.27) Q4: 0.90 (0.43; 1.87) Q5: 1.91 (0.98; 3.70)
Saber et al. 2017 [34]			
No. of cases	408		256
EPA in PL	HR Q2-4 vs. Q1: Q2: 1.03 (0.70; 1.52) Q3: 0.97 (0.65; 1.44) Q4: 1.06 (0.71; 1.59)		HR Q2-4 vs. Q1: Q2: 1.10 (0.72; 1.66) Q3: 0.96 (0.62; 1.50) Q4: 0.94 (0.61; 1.45)
DPA in PL	HR for Q2-4 vs. Q1: Q2: 1.05 (0.72; 1.53) Q3: 1.02 (0.69; 1.53) Q4: 0.98 (0.64; 1.53)		HR Q2-4 vs. Q1: Q2: 0.75 (0.50; 1.14) Q3: 0.83 (0.55; 1.35) Q4: 0.59 (0.38; 0.93)
DHA in PL	HR Q2-4 vs. Q1: Q2: 0.88 (0.60; 1.16) Q3: 0.72 (0.48; 1.07) Q4: 0.53 (0.34; 0.83)		HR Q2-4 vs. Q1: Q2: 1.25 (0.83; 1.90) Q3: 1.07 (0.69; 1.65) Q4: 1.09 (0.69; 1.72)
Venø et al. 2019 [35]			
No. of cases in dietary analyses	319	844	102
No. of cases in AT analyses	300	781	99
Total LC n-3 intake	HR Q2-4 vs. Q1: Q2: 0.97 (0.72; 1.30) Q3: 0.88 (0.65; 1.19) Q4: 0.69 (0.50; 0.95)	HR Q2-4 vs. Q1: Q2: 1.15 (0.94; 1.40) Q3: 1.20 (0.98; 1.45) Q4: 1.06 (0.87: 1.30)	HR Q2-4 vs. Q1: Q2: 1.36 (0.69; 2.66) Q3: 1.49 (0.78; 2.88) Q4: 2.50 (1.38; 4.53)
Total LC n-3 in AT	HR Q2-4 vs. Q1: Q2: 0.86 (0.61; 1.22) Q3: 1.09 (0.78; 1.52) Q4: 0.78 (0.53; 1.13)	HR Q2-4 vs. Q1: Q2: 0.91 (0.72; 1.15) Q3: 1.05 (0.83; 1.32) Q4: 0.99 (0.79; 1.26)	HR Q2-4 vs. Q1: Q2: 2.08 (1.04; 4.15) Q3: 2.04 (1.03; 4.04) Q4: 2.63 (1.33; 5.19)
EPA intake	HR Q2-4 vs. Q1: Q2: 0.86 (0.64; 1.16) Q3: 0.82 (0.60; 1.11) Q4: 0.66 (0.48; 0.91)	HR Q2-4 vs. Q1: Q2: 1.14 (0.94; 1.38) Q3: 1.16 (0.96; 1.41) Q4: 1.05 (0.86; 1.28)	HR Q2-4 vs. Q1: Q2: 1.17 (0.58; 2.35) Q3: 2.34 (1.27; 4.30) Q4: 2.02 (1.09; 3.73)
EPA in AT	HR Q2-4 vs. Q1: Q2: 0.96 (0.70; 1.32) Q3: 0.64 (0.43; 0.94) Q4: 0.52 (0.36; 0.76)	HR Q2-4 vs. Q1: Q2: 0.84 (0.68; 1.04) Q3: 0.61 (0.47; 0.79) Q4: 0.69 (0.55; 0.88)	HR Q2-4 vs. Q1: Q2: 1.13 (0.61; 2.11) Q3: 1.06 (0.52; 2.14) Q4: 1.52 (0.82; 2.81)
DPA intake	HR Q2-4 vs. Q1: Q2: 1.01 (0.74; 1.38) Q3: 0.91 (0.67; 1.25) Q4: 0.88 (0.64; 1.21)	HR Q2-4 vs. Q1: Q2: 1.27 (1.04; 1.55) Q3: 1.21 (0.99; 1.48) Q4: 1.19 (0.98; 1.46)	HR Q2-4 vs. Q1: Q2: 0.80 (0.41: 1.56) Q3: 1.14 (0.63; 2.08) Q4: 1.68 (0.96; 2.91)
DPA in AT	HR Q2-4 vs. Q1: Q2: 1.46 (1.04; 2.07) Q3: 1.23 (0.86; 1.76) Q4: 1.02 (0.69; 1.51)	HR Q2-4 vs. Q1: Q2: 1.37 (1.08; 1.74) Q3: 1.29 (1.01; 1.64) Q4: 1.19 (0.93; 1.52)	HR Q2-4 vs. Q1: Q2: 2.45 (1.19; 5.05) Q3: 2.61 (1.30; 5.25) Q4: 3.06 (1.50; 6.24)
DHA intake	HR Q2-4 vs. Q1: Q2: 0.90 (0.67; 1.22) Q3: 0.86 (0.63; 1.16) Q4: 0.72 (0.53; 0.99)	HR Q2-4 vs. Q1: Q2: 1.26 (1.04; 1.54) Q3: 1.17 (0.96; 1.43) Q4: 1.13 (0.93; 1.38)	HR Q2-4 vs. Q1: Q2: 0.97 (0.50; 1.89) Q3: 1.27 (0.68; 2.36) Q4: 2.12 (1.21; 3.69)
DHA in AT	HR Q2-4 vs. Q1: Q2: 0.94 (0.67; 1.32) Q3: 1.08 (0.77; 1.52) Q4: 0.79 (0.54; 1.16)	HR Q2-4 vs. Q1: Q2: 0.86 (0.68; 1.09) Q3: 1.07 (0.85; 1.34) Q4: 0.92 (0.72; 1.17)	HR Q2-4 vs. Q1: Q2: 1.37 (0.71; 2.64) Q3: 1.64 (0.87; 3.10) Q4: 2.00 (1.04; 3.84)
Venø et al. 2017 [36]			
No. of cases	319	844	102
LA intake	LA for SFA: HR: 0.84 (0.57; 1.25) LA for MUFA: HR: 1.05 (0.58; 1.90) LA for glycemic carbohydrates: HR: 0.96 (0.64; 1.44)	LA for SFA: HR: 0.96 (0.75; 1.23) LA for MUFA: HR: 0.67 (0.46; 0.96) LA for glycemic carbohydrates: HR: 0.82 (0.64; 1.05)	LA for SFA: HR: 1.46 (0.75; 2.85) LA for MUFA: HR: 1.35 (0.51; 3.55) LA for glycemic carbohydrates: HR: 1.55 (0.81; 3.00)
Venø et al. 2018 [37]			
No. of cases	300	781	99
LA in AT	HR Q2-4 vs. Q1: Q2: 0.72 (0.51; 1.01) Q3: 0.84 (0.61; 1.17) Q4: 0.61 (0.43; 0.88)	HR Q2-4 vs. Q1: Q2: 0.90 (0.72; 1.13) Q3: 0.87 (0.69; 1.03) Q4: 0.87 (0.69; 1.11)	HR Q2-4 vs. Q1: Q2: 1.28 (0.75; 2.19) Q3: 0.71 (0.37; 1.37) Q4: 0.86 (0.46; 1.59)

Abbreviations: NS, Not statistically significant; AT, Adipose tissue. ^1^ Univariate analysis. ^2^ 99% Confidence interval. The presented measures of association was selected for models that included adjustment for lifestyle factors without including dietary covariates whenever possible. See Table 1 and Tables 3–7 for study characteristics.

**Table 3:** Characteristics of observational follow-up and nested case-control studies investigating biomarkers of alpha-linolenic acid (ALA) and total ischemic stroke.

Author and Study	Study Population, Design and Country	Sex	No. of Partici-pants and Cases	Baseline Age (yrs)	Biomarker	Concentrations (%)	Follow-Up (yrs)	Measure of Association (95% CI)	Variables Controlled for
Iso et al. 2002 [31]	A Japanese cardiovascular risk survey population; Nested case-control; Japan	M+W	122 cases & 366 controls	40–85	ALA in serum	Cases: 1.0% Controls: 1.0%	Not reported	OR: 0.86 (NS, CI not reported) per 1 SD increase in ALA	Univariate analysis. Matched by age; sex; community; year of serum storage; fasting status.
De Goede et al. 2013 [38]	The MORGEN Study cohort; Nested-case control; The Netherlands	M+W	93 cases & 93 controls	20–65	ALA in CE	Cases: 0.53% Controls: 0.52%	10.5	OR: 1.02 (0.71; 1.46) for 1 SD increase in ALA	Age; gender; enrollment date; smoking; BMI; alcohol; high educational level; diabetes; hypertension; hypercholesterolemia.
Yamagishi et al. 2013 [39]	The Minneapolis field center of the ARIC cohort; Follow-up; United States	M+W	3870 subjects including 168 cases	45–65	ALA in PL and CE, separately	Mean: CE: 0.4% PL: 0.1%	19.9	HR for Q4 vs. Q1: CE: 1.14 (0.76; 1.72) PL: 1.29 (0.82; 2.02)	Age and sex. The authors stated that the point estimated did not materially change after additional adjustment for smoking status; cigarette-years and alcohol intake.
Yaemsiri et al. 2013 [32]	The WHI-OS cohort; Nested case-control; United status	W	964 cases & 964 controls	50–79	ALA in serum	Cases: 0.53% Controls: 0.55%	Not reported	OR: 0.90 (0.74; 1.08) for 1 SD increase in ALA ^2^	Age; race; time of follow-up; BMI; smoking status; diabetes; aspirin use.
Fretts et al. 2014 [26]	The CHS cohort; Follow-up; United States	M+W	2709 subjects including 337 cases	≥65	ALA in PL	Q1: 0.09% Q3: 0.14% Q5: 0.22%	11.3 ^1^	Q2: 0.92 (0.65; 1.30) Q3: 1.01 (0.72; 1.43) Q4: 0.84 (0.59; 1.20) Q5: 0.97 (0.69; 1.36)	Age; sex; race; enrollment site; education; smoking status; diabetes; BMI; waist circumference; physical activity; alcohol; treated hypertension.
Daneshmand et al. 2016 [40]	The KIHD cohort; Follow-up; Finland	M	1828 subjects including 153 cases	42–60	ALA in serum	Q1: <0.58% Q4: >0.87%	21.2	Q2: 0.89 (0.58; 1.36) Q3: 0.63 (0.39; 1.02) Q4: 0.90 (0.58; 1.41)	Age; examination year; BMI; smoking status; physical activity; alcohol.
Bork et al. 2018 [33]	The DCH cohort; Case-cohort; Denmark	M+W	54,648 subjects including 1735 cases	50–65	ALA in adipose tissue	Q1: 0.64% Q3: 0.84% Q5: 1.05%	13.4	Q2: 0.95 (0.78; 1.16) Q3: 0.86 (0.70; 1.06) Q4: 0.93 (0.76; 1.14) Q5: 1.01 (0.82; 1.23)	Age; sex; duration of schooling; smoking; physical activity; waist circumference adjusted for BMI; alcohol.

Abbreviations: M, men; W, Women; OR, odds ratio; CI, Confidence interval; NS, not statistically significant; SD, standard deviation; MORGEN, The Monitoring Project on Risk Factors for Chronic Diseases; CE, Cholesterol ester; BMI, Body mass index; ARIC, Atherosclerosis Risk in Communities; PL, Plasma phospholipids; HR, Hazard ratio; Q, Quintile; CHS, Cardiovascular Health Study; KIHD, The Kuopio, Ischaemic Heart Disease Risk Factor Study; WHI-OS, The Women’s Health Initiative Observational Follow-up Study; DCH, Diet, Cancer and Health. The presented measures of association was selected for models that included adjustment for lifestyle factors without including dietary covariates whenever possible. The lowest group of exposure was used as reference unless otherwise indicated. ^1^ Follow-up time was calculated as person-years of follow-up divided by the total number of participants; ^2^ 99% confidence interval.

**Table 4:** Characteristics of observational follow-up studies investigating intake of LC n-3 PUFAs and total ischemic stroke.

Author and Study	Study Population Country	Sex	No. of Participants	Baseline Age (yrs)	Exposure Assessment	Intake (mg/d)	No. of Cases	Follow-up (yrs)	Hazard Ratios (95% CI)	Variables Controlled for
Iso et al. 2001 [30]	The NHS cohort; United States	W	79,839	34–59	FFQ	EPA+DHA Q1: 77 Q3: 171 Q5: 481	303	14	Q2: 0.83 (0.59; 1.18) Q3: 0.67 (0.47; 0.98) Q4: 0.82 (0.57; 1.18) Q5: 0.71 (0.46; 1.10)	Age; smoking status; time interval; joules; BMI; alcohol; menopausal status; post-menopausal hormone use; vigorous exercise; aspirin; multivitamins; hypertension; fruit; vegetables; SFA; trans unsaturated fat; linoleic acid; animal protein; calcium.
He et al. 2002 [41]	The HPFS cohort; United States	M	43,671	40–75	FFQ	EPA+DHA Q1: <50 Q3: 200–400 Q5: ≥600	377	12	Q2: 0.56 (0.35; 0.88) Q3: 0.63 (0.40; 0.98) Q4: 0.54 (0.32; 0.91) Q5: 0.73 (0.43; 1.25)	Age; smoking status; BMI; physical activity; hypertension; aspirin; fish oil; multivitamins; total calories; total fat; SFA; trans-unsaturated fat; alcohol; potassium; magnesium; fruit; vegetables; hypercholesterolemia.
Yamagishi et al. 2008 [46]	The JACC cohort, Japan	M+W	57,972	40–79	FFQ	Total LCn-3 Q1: <1180 Q5: ≥2110	319	12.7	Q2: 1.06 (0.69; 1.63) Q3: 1.31 (0.85; 2.01) Q4: 1.07 (0.68; 1.69) Q5: 1.17 (0.71; 1.92)	Age; sex; hypertension; diabetes; smoking status; alcohol; BMI; mental stress; walking; sports; education levels; total energy; intake of cholesterol, SFA, n-6 PUFAs, vegetables; fruit.
Wallström et al. 2011 [45]	The Malmö Diet and Cancer cohort, Sweden	M & W	M: 8083 W: 12,402	44–73	Combined FFQ, 7-day diet history and 1 hour diet interview	EPA+DHA M: Q1: 0.08% ^1^ Q3: 0.19% ^1^ Q5: 0.53% ^1^ W: Q1: 0.07% ^1^ Q3: 0.18% ^1^ Q5: 0.49% ^1^	M: 397 W: 346	13.5	M: Q2: 1.36 (0.97; 1.89) Q3: 1.29 (0.92; 1.80) Q4: 1.12 (0.80; 1.57) Q5: 1.12 (0.80; 1.57) W: Q2: 0.87 (0.60; 1.26) Q3: 0.98 (0.68; 1.40) Q4: 0.98 (0.69; 1.41) Q5: 1.04 (0.73; 1.48)	Age; diet assessment method version; total energy intake; season; BMI; smoking; education; alcohol; systolic blood pressure, anti-hypertensive treatment; anti-hyperlipidemic treatment; leisure time physical activity; dietary fiber.
Montonen et al. 2009 [43]	The Finnish Mobile Clinic Health Examination Survey cohort, Finland	M+W	3958	40–79	Dietary history interview	EPA+DHA Q1: 102 Q4: 655	364	28	Q2: 0.95 (0.71; 1.28 Q3: 0.99 (0.74; 1.33) Q4: 0.91 (0.66; 1.26)	Age; sex; energy intake; smoking; BMI; physical activity; geographic area; occupation; diabetes; post-hormonal hormone use; hypertension; S-cholesterol; intake of butter, vegetables, fruit and berries.
Larsson et al. 2012 [28]	The Swedish Mammography cohort; Sweden	W	34,670	49–83	FFQ	EPA+DHA Q1: 131 Q3: 289 Q5: 559	1310	10.4	Q2: 0.88 (0.74; 1.04) Q3: 0.84 (0.70; 1.01) Q4: 0.83 (0.69; 0.99) Q5: 0.83 (0.69; 0.99)	Age; smoking status and pack-years of smoking; education; BMI; physical activity; hypertension; diabetes; aspirin; family history of MI; intakes of alcohol, protein, fiber and cholesterol.
De Goede et al. 2012 [42]	The MORGEN Study cohort; The Netherlands	M & W	M: 8,988W: 11,081	20–65	FFQ	EPA+DHA M: Q1: 44 Q4: 241 W: Q1: 36 Q4: 225	M: 80 W: 64	10.5	M: Q2: 0.93 (0.50; 1.74) Q3: 0.87 (0.46; 1.65) Q4: 0.85 (0.45; 1.60) W: Q2: 0.98 (0.50; 1.91) Q3: 0.98 (0.50; 1.93) Q4: 0.62 (0.29; 1.35)	Age; smoking; BMI; educational level; parental history of MI; alcohol; total energy intake; intake of fiber, vitamin C, beta-carotene, SFAs, trans fatty acids, MUFAs, linoleic acid and ALA.
Kippler et al. 2016 [44]	The Cohort of Swedish Men, Sweden	M	39,948	45–79	FFQ	EPA+DHA Q1:180 Q4: 730	2286	12	Q2: 1.02 (0.90; 1.16) Q3: 0.98 (0.85; 1.13) Q4: 1.09 (0.94; 1.26)	Age; educational level; family history of MI; high cholesterol; hypertension; atrial fibrillation; aspirin; BMI; smoking status; alcohol; physical activity; fish oil supplements; energy intake; intake of fruit, vegetables, red and processed meat, dairy products, SFAs, MeHg and PCB.
Rhee et al. 2017 [27]	The WHS cohort; United States	W	38,392	≥45	FFQ	EPA+DHA Q1: 0.06% ^1^ Q3: 0.16% ^1^ Q5: 0.40% ^1^	807	18.2 ^2^	Q2: 0.99 (0.80; 1.23) Q3: 0.90 (0.72; 1.14) Q4: 0.92 (0.74; 1.14) Q5: 1.04 (0.84; 1.29)	Age; randomized treatment assignment; BMI; smoking; alcohol; physical activity; oral conceptive use; HRT; multivitamin use; energy intake; family history of MI; hypertension; high cholesterol; diabetes.
Venø et al. 2018 [35]	The DCH cohort; Denmark	M+W	55,338	50–65	FFQ	Total LCn-3 median: ~700	1879	13.5	Q2: 1.06 (0.93; 1.21) Q3: 1.06 (0.93; 1.21) Q4: 1.06 (0.93; 1.20)	Age; sex; education; waist circumference adjusted by BMI; smoking; physical activity; alcohol; alcohol abstain.

Abbreviations: NHS, Nurses’s Health Study; W, Women; FFQ, Food Frequency Questionnaire; EPA, Eicosapentaenoic acid; DHA, Docosahexaenoic acid; Q, Quintile; CI, Confidence interval; BMI, Body mass index; SFA, Saturated fatty acids; M, men; HPFS, Health Professionals Follow-up Study; JACC, Japan Collaborative Cohort Study for Evaluation of Cancer Risk Study; LCn-3, Long-chain n-3 PUFAs; MI, Myocardial infarction; MORGEN, Monitoring Project on Risk Factors for Chronic Diseases; MUFA, Monounsaturated fatty acids; MeHg, Methylmercery; PCB, Polychlorinated biphenyls; WHS, Women’s Health Study; DCH, Diet, Cancer and Health. The presented measures of association was selected for models that included adjustment for lifestyle factors without including dietary covariates whenever possible. The lowest group of exposure was used as reference unless otherwise indicated. ^1^ Percentage of total energy intake; ^2^ Follow-up time was calculated as person years divided by the total number of participants.

**Table 5:** Characteristics of observational follow-up and nested case-control studies investigating biomarkers of LC n-3 PUFAs and total ischemic stroke.

Author and Study	Study Population, Design and Country	Sex	No. of Partici-pants and Cases	Baseline Age (yrs)	Biomarker	Concentrations (%)	Follow-up (yrs)	Measure of Association (95% CI)	Variables Controlled for
Iso et al. 2002 [31]	A Japanese cardio-vascular risk survey population; Nested case-control; Japan	M+W	122 cases & 366 controls	40–85	EPA, DPA and DHA in serum (separately)	EPA: Cases: 3.6 Controls: 3.7 DHA: Cases: 4.5 Controls: 4.4	Not reported	OR: 1.01 (NS, CI not reported) per 1 SD increase in EPA OR: 1.03 (NS, CI not reported) per 1 SD increase in DHA	Univariate analysis. Matched by age; sex; community; year of serum storage; fasting status.
De Goede et al. 2013 [38]	The MORGEN Study cohort; Nested-case control; The Netherlands	M+W	93 cases & 93 controls	20–65	EPA+DHA in CE	Cases: 1.57 Controls: 1.25	10.5	OR: 1.33 (0.96; 1.84) for 1 SD increase in EPA+DHA	Age; gender; enrollment date; smoking; BMI; alcohol; high educational level; diabetes; hypertension; hypercholesterolemia.
Mozaffarian et al. 2013 [48]	The CHS cohort; Follow-up; United States	M+W	2692 subjects including 319 cases	≥65	Total LCn-3, EPA, DPA and DHA in PL	Total LCn-3 Q1: 3.17 Q3: 4.21 Q5: 6.04	11.5 ^1^	Q2: 0.88 (0.63; 1.23) Q3: 0.77 (0.54; 1.08) Q4: 0.93 (0.66; 1.31) Q5: 0.63 (0.43; 0.94)	Age; sex; race; enrollment site; fatty acid measurement batch; education; smoking status; diabetes; atrial fibrillation; treated hypertension; leisure time physical activity; BMI; waist circumference; alcohol; intake of broiled and baked fish, fried fish, red meat, fruits, vegetables and fiber.
Yaemsiri et al. 2013 [32]	The WHI-OS cohort; Nested case-control; United status	W	964 cases & 964 controls	50–79	EPA, DPA and DHA in serum (separately)	EPA: Cases: 0.56 Controls: 0.60 DHA: Cases: 1.75 Controls: 1.97	Not reported	OR: 0.84 (0.70; 1.01) for 1 SD increase in EPA ^2^ OR: 0.72 (0.59; 0.87) for 1 SD increase in DHA ^2^	Age; race; time of follow-up; BMI; smoking status; diabetes; aspirin.
Yamagishi et al. 2013 [39]	The Minneapolis field center of the ARIC cohort; Follow-up; United States	M+W	3870 subjects including 168 cases	45–65	Total LCn-3, EPA and DHA in PL and CE (separately)	Total LCn-3 CE: Q1: 0.22–0.77 Q4: 1.15–6.02 PL: Q1: 1.51–3.57 Q4: 4.75–13.5	19.9	HR for Q4 vs. Q1: CE: 1.16 (0.75; 1.79) PL: 0.86 (0.56; 1.32)	Age and sex. The authors stated that the point estimated did not materially change after additional adjustment for smoking status; cigarette-years and alcohol intake.
Daneshmand et al. 2016 [40]	The KIHD cohort; Follow-up; Finland	M	1828 subjects including 153 cases	42–60	Total LCn-3, EPA, DPA and DHA in serum	Total LCn-3 Q1: <3.63% Q4: >5.34%	21.2	Q2: 0.66 (0.41; 1.07) Q3: 0.91 (0.58; 1.42) Q4: 0.98 (0.64; 1.51)	Age; examination year; BMI; smoking status; physical activity; alcohol.
Saber et al. 2017 [34]	Pooled analysis of the NHS and HFPS (nested case-control), and the CHS (follow-up) cohorts, United States	M+W	A total of 953 cases and 437 controls from the NHS and HFPS and 3941 subjects from the CHS	NHS: 30–55 HFPS: 40–75 CHS: ≥65	EPA, DPA and DHA in PL (separately)	Cohort-specific fatty acid content in quartiles can be found in the paper.	NHS+HPFS: 11.2 CHS: 8.3	EPA: Q2: 0.95 (0.77; 1.18) Q3: 0.93 (0.74; 1.16) Q4: 0.94 (0.77; 1.19) DHA: Q2: 0.93 (0.75; 1.14) Q3: 0.85 (0.71; 1.10) Q4: 0.80 (0.63; 1.00)	Age; race; sex; smoking status; physical activity; alcohol; hypertension; family history of diabetes; parental history of CVD; menopausal status; postmenopausal hormone use; BMI; aspirin; intake of processed and unprocessed meat, fruits and vegetables.
Venø et al. 2019 [35]	The DCH cohort; Case-cohort, Denmark	M+W	55,338 subjects including 1755 cases	50–65	Total LCn-3, EPA, DPA and DPA in adipose tissue	Total LCn-3: Q1: 0.42 Q4: 0.94	13.5	Q2: 0.98 (0.82; 1.17) Q3: 1.12 (0.94; 1.33) Q4: 1.08 (0.90; 1.30)	Age; sex; education; waist circumference adjusted for BMI; smoking; physical activity; alcohol and alcohol abstain.

Abbreviations: M, men; W, Women; EPA, Eicosapentaenoic acid; DPA, Docosapentaenoic acid; DHA, Docosahexaenoic acid; OR, Odds ratio; CI, Confidence interval; NS, Not statistically significant; SD, Standard deviation; MORGEN, The Monitoring Project on Risk Factors for Chronic Diseases; CE, Cholesterol esters; BMI, Body mass index; CHS, Cardiovascular Health Study; LC n-3, Long-chain n-3 PUFAs, PL, Plasma phospholipids; Q, Quintile; WHI-OS, Women’s Health Initiative Observational Follow-up Study; KIHD, The Kuopio, Ischaemic Heart Disease Risk Factor Study; NHS, Nurses’ Health Study; HPFS, Health Professionals Follow-up Study; DCH, Diet, Cancer and Health. The presented measures of association was selected for models that included adjustment for lifestyle factors without including dietary covariates whenever possible. The lowest group of exposure was used as reference unless otherwise indicated. ^1^ Follow-up time was calculated as person years divided by the total number of participants; ^2^ 99% confidence interval.

**Table 6:** Characteristics of observational follow-up studies investigating intake of linoleic acid (LA) and total ischemic stroke.

Author and Study	Study Population and Country	Sex	No. of Participants	Baseline Age (yrs)	Exposure Assessment	Intake (g/d)	No. of Cases	Follow-up (yrs)	Hazard Ratios (95% CI)	Variables Controlled for
Larsson et al. 2012 [28]	The Swedish Mammography Cohort, Sweden	W	34,670	49–83	FFQ	LA+AA Q1: 4.7 Q3: 6.0 Q5: 8.0	1310	10.4	Q2: 0.98 (0.84; 1.16) Q3: 0.96 (0.81; 1.14) Q4: 1.02 (0.85; 1.21) Q5: 0.96 (0.80; 1.15)	Age; smoking status and pack-years of smoking; education; BMI; physical activity; hypertension; diabetes; aspirin; family history of MI; intakes of alcohol, protein, fiber and cholesterol.
Wallstrøm et al. 2012 [45]	The Malmö Diet and Cancer cohort, Sweden	M & W	M: 8083 W: 12,402	44–73	Combined FFQ, 7-day diet history and 1 h diet interview	LA+AA: Q1: 3.5% ^1^ Q3: 5.0% ^1^ Q5: 7.1% ^1^ W: Q1: 3.3% ^1^ Q3: 4.7% ^1^ Q5: 6.7% ^1^	M: 397W: 346	13.5	M: Q2: 1.12 (0.82; 1.52) Q3: 1.02 (0.74; 1.39) Q4: 1.10 (0.80; 1.50) Q5: 1.16 (0.84; 1.58) W: Q2: 0.99 (0.72; 1.36) Q3: 0.86 (0.62; 1.19) Q4: 1.02 (0.73; 1.41) Q5: 0.81 (0.57; 1.14)	Age; diet assessment method version; total energy intake; season; BMI; smoking; education; alcohol; systolic blood pressure, anti-hypertensive treatment; anti-hyperlipidemic treatment; leisure time physical activity; dietary fiber.
Venø et al. 2017 [36]	The DCH cohort; Denmark	M+W	55,338	50–65	FFQ	Median 10.7	1879	13.5	LA for SFA: 0.98 (0.83; 1.16) LA for MUFA 0.80 (0.63; 1.02) LA for glycemic carbohydrates: 0.92 (0.78; 1.09)	Age; sex; total energy intake; education status; physical activity; waist circumference adjusted for BMI; alcohol; alcohol abstain; smoking.

Abbreviations: M, men; W, Women; FFQ, Food Frequency Questionnaire; AA, Arachidonic acid; Q, Quintile; CI, Confidence interval; BMI, Body mass index; MI, Myocardial infarction; SFA, Saturated fatty acids; MUFA, Monounsaturated fatty acids; DCH, Diet Cancer and Health. The presented measures of association was selected for models that included adjustment for lifestyle factors without including dietary covariates whenever possible. The lowest group of exposure was used as reference unless otherwise indicated. ^1^ Percentage of total energy intake.

**Table 7:** Characteristics of observational follow-up and nested case-control studies investigating biomarkers of linoleic acid (LA) and total ischemic stroke.

Author and Study	Study Population, Design and Country	Sex	No. of Partici-pants and Cases	Baseline Age (yrs)	Biomarker	Concentrations (%)	Follow-up (yrs)	Measure of Association (95% CI)	Variables Controlled for
Iso et al. 2002 [31]	A Japanese cardiovascular risk survey population; Nested case-control; Japan	M+W	122 cases & 366 controls	40–85	LA in serum	Cases: 26.1% Controls: 27.9%	Not reported	OR 0.66 (0.49; 0.88) per 1 SD increase in LA	Age; sex; community; year of serum storage; fasting status; BMI; cigarette smoking status; alcohol; hypertension; serum cholesterol levels, triglyceride and glucose levels.
Yaemsiri et al. 2013 [32]	The WHI-OS cohort; Nested case-control; United status	W	964 cases &964 controls	50–79	LA in serum	Cases: 26.7% Controls: 27.1%	Not reported	OR: 0.92 (0.79; 1.08) for 1 SD increase in LA ^2^	Age; race; time of follow-up; BMI; smoking status; diabetes; aspirin.
Yamagishi et al. 2013 [39]	The Minneapolis field center of the ARIC cohort; Follow-up; United States	M+W	3870 subjects including 168 cases	45–65	LA in PL and CE (separately)	CE: Q1: 21.5–51.3% Q4: 57.4–68.2% PL: Q1: 9.0–20.3% Q4: 23.7–32.4%	19.9	HR for Q4 vs. Q1: CE: 0.64 (0.43; 0.97) PL: 0.69 (0.45; 1.05)	Age and sex. The authors stated that the point estimated did not materially change after additional adjustment for smoking status; cigarette-years and alcohol intake.
De Goede et al. 2013 [38]	The MORGEN Study cohort; Nested-case control; The Netherlands	M+W	93 cases & 93 controls	20–65	LA in CE	Cases: 54.2% Controls: 55.4%	10.5	OR: 0.81 (0.54; 1.24) for 1 SD increase in LA	Age; gender; enrollment date; smoking; BMI; alcohol; high educational level; diabetes; hypertension; hypercholesterolemia.
Wu et al. 2014 [49]	The CHS cohort, Follow-up, United States	M+W	2792 subjects including 362 cases	≥65	LA in PL	Q1: 16.6% Q3: 19.7% Q5: 22.9%	12.3 ^1^	Q2: 1.00 (0.71; 1.40) Q3: 0.90 (0.64; 1.27) Q4: 0.88 (0.62; 1.26) Q5: 0.88 (0.61; 1.27)	Age; sex; race; enrollment site; education; smoking status; diabetes; atrial fibrillation; hypertension; leisure-time physical activity; BMI; waist circumference; alcohol; plasma LC n-3 PUFAs.
Daneshmand et al. 2016 [40]	The KIHD cohort; Follow-up; Finland	M	1828 subjects including 153 cases	42–60	LA in serum	Q1: <23.7% Q4: >29.5%	21.2	Q2: 0.72 (0.46; 1.14) Q3: 0.87 (0.55; 1.37) Q4: 1.07 (0.68; 1.67)	Age; examination year; BMI; smoking status; physical activity; alcohol.
Venø et al. 2018 [37]	The DCH cohort, Case-cohort, Denmark	M+W	55,338 subjects including 1755 cases	50–65	LA in adipose tissue	Q1: <9.6% Q4: >11.7%	13.5	Q2: 0.92 (0.77; 1.09) Q3: 0.85 (0.71; 1.02) Q4: 0.78 (0.65; 0.93)	Age; sex; alcohol; alcohol abstain; waist circumference adjusted for BMI; education status; smoking; physical activity.
Marklund et al. 2019 [23]	FORCE Consortium; Individual pooled analysis of cohort studies Multinational	M+W	The FORCE Consortium population including 3705 cases	49–77	LA in PL, TP; CE, & AT	PL: 6.9% TP: 11.4% CE: 11.9% RBC: 4.9% AT: 6.2%	2.5–31.9	HR per IQR: PL: 0.95 (0.82; 1.10) TP: 0.84 (0.66; 1.06) CE: 0.67 (0.51; 0.88) AT: 0.87 (0.65; 1.15) Overall: 0.88 (0.79; 0.98)	Age; sex; race; field center; BMI; education; smoking; physical activity; alcohol; diabetes; treated hypertension; treated hypercholesterolemia; aspirin; levels of ALA, EPA and trans isomers of oleic acid and trans isomers of linoleic acid.

Abbreviations: M, men; W, Women; OR, odds ratio; SD, standard deviation; BMI, Body mass index; WHI-OS, Women’s Health Initiative Observational Follow-up Study; ARIC, Atherosclerosis Risk in Communities; CE, Cholesterol ester; PL, Phospholipids; HR, Hazard ratio; CI, Confidence interval; Q, Quintile; MORGEN, Monitoring Project on Risk Factors for Chronic Diseases; CHS, Cardiovascular Health Study; LC n-3, Long-chain n-3 polyunsaturated fatty acids; KIHD, Kuopio, Ischaemic Heart Disease Risk Factor Study; DCH, Diet, Cancer and Health; FORCE, Fatty Acid and Outcomes Research Consortium; TP, Total plasma; AT, Adipose tissue; ALA, alpha-linolenic acid; EPA, Eicosapentanoic acid. The presented measures of association was selected for models that included adjustment for lifestyle factors without including dietary covariates whenever possible. The lowest group of exposure was used as reference unless otherwise indicated. ^1^ Follow-up time was calculated as person-years of follow-up divided by the total number of participants; ^2^ 99% confidence interval.

## References

[1] Sacco R., Kasner S., Broderick J., Caplan L., Connors J., Culebras A., Elkind M., George M., Hamdan A., Higashida R. (2013). An Updated Definition of Stroke for the 21st Century. Stroke.

[2] Hansen C., Overvad K., Kyrø C., Olsen A., Tjønneland A., Johnsen S., Jakobsen M., Dahm C. (2017). Adherence to a Healthy Nordic Diet and Risk of Stroke: A Danish Cohort Study. Stroke.

[3] Chiuve S., Rexrode K., Spiegelman D., Logroscino G., Manson J., Rimm E. (2008). Primary prevention of stroke by healthy lifestyle. Circulation.

[4] De Caterina R. (2011). N-3 Fatty Acids in Cardiovascular Disease. N. Engl. J. Med..

[5] Baker E., Miles E., Burdge G., Yaqoob P., Calder P. (2016). Metabolism and functional effects of plant-derived omega-3 fatty acids in humans. Prog. Lipid Res..

[6] Raatz S.K., Conrad Z., Jahns L. (2018). Trends in linoleic acid intake in the United States adult population: NHANES 1999-2014. Prostaglandins Leukot. Essent. Fatty Acids.

[7] Whelan J., Fritsche K. (2013). Linoleic acid. Adv. Nutr..

[8] Gebauer S., Psota T., Harris W., Kris-Etherton P. (2006). n-3 Fatty acid dietary recommendations and food sources to achieve essentiality and cardiovascular benefits. Am. J. Clin. Nutr..

[9] Bork C., Jakobsen M., Lundbye-Christensen S., Tjønneland A., Schmidt E., Overvad K. (2016). Dietary intake and adipose tissue content of alpha-linolenic acid and risk of myocardial infarction: A Danish cohort study. Am. J. Clin. Nutr..

[10] Hu F., Stampfer M., Manson J., Rimm E., Wolk A., Colditz G., Hennekens C., Willett W. (1999). Dietary intake of alpha-linolenic acid and risk of fatal ischemic heart disease among women. Am. J. Clin. Nutr..

[11] Burdge G. (2018). Is essential fatty acid interconversion an important source of polyunsaturated fatty acids in humans?. Br. J. Nutr..

[12] Dyerberg J., Bang H., Stoffersen E., Moncada S., Vane J. (1978). Eicosapentaenoic acid and prevention of thrombosis and atherosclerosis?. Lancet.

[13] Dyerberg J., Schmidt E. (1989). n-3 fatty acids and cardiovascular disease -observations generated by studies in Greenland Eskimos. Wien. Klin. Wochenschr..

[14] Schmidt E. (1997). n-3 fatty acids and the risk of coronary heart disease. Dan. Med. Bull..

[15] Calder P. (2015). Marine omega-3 fatty acids and inflammatory processes: Effects, mechanisms and clinical relevance. Biochim. Biophys. Acta.

[16] Mozaffarian D., Wu J. (2011). Omega-3 fatty acids and cardiovascular disease: Effects on risk factors, molecular pathways, and clinical events. J. Am. Coll. Cardiol..

[17] Saravanan P., Davidson N., Schmidt E., Calder P. (2010). Cardiovascular effects of marine omega-3 fatty acids. Lancet (London, England).

[18] Rajaram S. (2014). Health benefits of plant-derived alpha-linolenic acid. Am. J. Clin. Nutr..

[19] Wang D. (2018). Dietary n-6 polyunsaturated fatty acids and cardiovascular disease: Epidemiologic evidence. Prostaglandins Leukot. Essent. Fatty Acids.

[20] Innes J., Calder P. (2018). Omega-6 fatty acids and inflammation. Prostaglandins Leukot. Essent. Fatty Acids.

[21] Bork C., Baker E., Lundbye-Christensen S., Miles E., Calder P. (2019). Lowering the linoleic acid to alpha-linoleic acid ratio decreases the production of inflammatory mediators by cultured human endothelial cells. Prostaglandins Leukot. Essent. Fatty Acids.

[22] Alexander D., Miller P., Van Elswyk M., Kuratko C., Bylsma L. (2017). A Meta-Analysis of Randomized Controlled Trials and Prospective Cohort Studies of Eicosapentaenoic and Docosahexaenoic Long-Chain Omega-3 Fatty Acids and Coronary Heart Disease Risk. Mayo Clin. Proc..

[23] Marklund M., Wu J., Imamura F., Del Gobbo L., Fretts A., de Goede J., Shi P., Tintle N., Wennberg M., Aslibekyan S. (2019). Biomarkers of Dietary Omega-6 Fatty Acids and Incident Cardiovascular Disease and Mortality: An Individual-Level Pooled Analysis of 30 Cohort Studies. Circulation.

[24] Farvid M., Ding M., Pan A., Sun Q., Chiuve S., Steffen L., Willett W., Hu F. (2014). Dietary linoleic acid and risk of coronary heart disease: A systematic review and meta-analysis of prospective cohort studies. Circulation.

[25] De Goede J., Verschuren W., Boer J., Kromhout D., Geleijnse J. (2011). Alpha-linolenic acid intake and 10-year incidence of coronary heart disease and stroke in 20,000 middle-aged men and women in the Netherlands. PLoS ONE.

[26] Fretts A., Mozaffarian D., Siscovick D., Sitlani C., Psaty B., Rimm E., Song X., McKnight B., Spiegelman D., King I. (2014). Plasma phospholipid and dietary α-linolenic acid, mortality, CHD and stroke: The Cardiovascular Health Study. Br. J. Nutr..

[27] Rhee J., Kim E., Buring J., Kurth T. (2017). Fish Consumption, Omega-3 Fatty Acids, and Risk of Cardiovascular Disease. Am. J. Prev. Med..

[28] Larsson S., Virtamo J., Wolk A. (2012). Dietary fats and dietary cholesterol and risk of stroke in women. Atherosclerosis.

[29] Bork C., Venø S., Lundbye-Christensen S., Jakobsen M., Tjønneland A., Schmidt E., Overvad K. (2018). Dietary Intake of α-Linolenic Acid Is Not Appreciably Associated with Risk of Ischemic Stroke among Middle-Aged Danish Men and Women. J. Nutr..

[30] Iso H., Rexrode K., Stampfer M., Manson J., Colditz G., Speizer F., Hennekens C., Willett W. (2001). Intake of fish and omega-3 fatty acids and risk of stroke in women. JAMA.

[31] Iso H., Sato S., Umemura U., Kudo M., Koike K., Kitamura A., Imano H., Okamura T., Naito Y., Shimamoto T. (2002). Linoleic acid, other fatty acids, and the risk of stroke. Stroke.

[32] Yaemsiri S., Sen S., Tinker L., Robinson W., Evans R., Rosamond W., Wasserthiel-Smoller S., He K. (2013). Serum fatty acids and incidence of ischemic stroke among postmenopausal women. Stroke.

[33] Bork C., Venø S., Lundbye-christensen S., Jakobsen M., Tjønneland A., Calder P., Overvad K., Schmidt E. (2018). Adipose tissue content of alpha-linolenic acid and the risk of ischemic stroke and ischemic stroke subtypes: A Danish case-cohort study. PLoS ONE.

[34] Saber H., Yakoob M., Shi P., Longstreth W., Lemaitre R., Siscovick D., Rexrode K., Willett W., Mozaffarian D. (2017). Omega-3 Fatty Acids and Incident Ischemic Stroke and Its Atherothrombotic and Cardioembolic Subtypes in 3 US Cohorts. Stroke.

[35] Venø S., Bork C., Jakobsen M., Lundbye-Christensen S., McLennan P., Bach F., Overvad K., Schmidt E. (2019). Marine n-3 Polyunsaturated Fatty Acids and the Risk of Ischemic Stroke. Stroke.

[36] Venø S., Schmidt E., Jakobsen M., Lundbye-Christensen S., Bach F., Overvad K. (2017). Substitution of Linoleic Acid for Other Macronutrients and the Risk of Ischemic Stroke. Stroke.

[37] Venø S., Bork C., Jakobsen M., Lundbye-Christensen S., Bach F., Overvad K., Schmidt E. (2018). Linoleic Acid in Adipose Tissue and Development of Ischemic Stroke: A Danish Case-Cohort Study. J. Am. Heart Assoc..

[38] De Goede J., Verschuren W., Boer J., Kromhout D., Geleijnse J. (2013). N-6 and n-3 fatty acid cholesteryl esters in relation to incident stroke in a Dutch adult population: A nested case-control study. Nutr. Metab. Cardiovasc. Dis..

[39] Yamagishi K., Folsom A., Steffen L. (2013). Plasma fatty acid composition and incident ischemic stroke in middle-aged adults: The Atherosclerosis Risk in Communities (ARIC) Study. Cerebrovasc. Dis..

[40] Daneshmand R., Kurl S., Tuomainen T., Virtanen J. (2016). Associations of serum n-3 and n-6 PUFA and hair mercury with the risk of incident stroke in men: The Kuopio Ischaemic Heart Disease Risk Factor Study (KIHD). Br. J. Nutr..

[41] He K., Rimm E., Merchant A., Rosner B., Stampfer M., Willett W., Ascherio A. (2002). Fish Consumption and Risk of Stroke in Men. JAMA.

[42] De Goede J., Verschuren W., Boer J.M., Kromhout D., Geleijnse J. (2012). Gender-specific associations of marine n-3 fatty acids and fish consumption with 10-year incidence of stroke. PLoS ONE.

[43] Montonen J., Järvinen R., Reunanen A., Knekt P. (2009). Fish consumption and the incidence of cerebrovascular disease. Br. J. Nutr..

[44] Kippler M., Larsson S., Berglund M., Glynn A., Wolk A., Åkesson A. (2016). Associations of dietary polychlorinated biphenyls and long-chain omega-3 fatty acids with stroke risk. Environ. Int..

[45] Wallström P., Sonestedt E., Hlebowicz J., Ericson U., Drake I., Persson M., Gullberg B., Hedblad B., Wirfält E. (2012). Dietary fiber and saturated fat intake associations with cardiovascular disease differ by sex in the Malmö Diet and Cancer Cohort: A prospective study. PLoS ONE.

[46] Yamagishi K., Iso H., Date C., Fukui M., Wakai K., Kikuchi S., Inaba Y., Tanabe N., Tamakoshi A. (2008). Fish, omega-3 polyunsaturated fatty acids, and mortality from cardiovascular diseases in a nationwide community-based cohort of Japanese men and women the JACC (Japan Collaborative Cohort Study for Evaluation of Cancer Risk) Study. J. Am. Coll. Cardiol..

[47] Bork C., Venø S., Lasota A., Lundbye-Christensen S., Schmidt E. (2019). Marine and plant-based n-3 PUFA and atherosclerotic cardiovascular disease. Proc. Nutr. Soc..

[48] Mozaffarian D., Lemaitre R., King I., Song X., Huang H., Sacks F., Rimm E., Wang M., Siscovick D. (2013). Plasma phospholipid long-chain ω-3 fatty acids and total and cause-specific mortality in older adults: A cohort study. Ann. Intern. Med..

[49] Wu J., Lemaitre R., King I., Song X., Psaty B., Siscovick D., Mozaffarian D. (2014). Circulating omega-6 polyunsaturated fatty acids and total and cause-specific mortality: The Cardiovascular Health Study. Circulation.

[50] Hodson L., Skeaff C., Fielding B. (2008). Fatty acid composition of adipose tissue and blood in humans and its use as a biomarker of dietary intake. Prog. Lipid Res..

[51] Arab L., Akbar J. (2002). Biomarkers and the measurement of fatty acids. Public Health Nutr..

[52] Adams H.J., Bendixen B., Kappelle L., Biller J., Love B., Gordon D., Marsh E. (1993). Classification of subtype of acute ischemic stroke. Definitions for use in a multicenter clinical trial. TOAST. Trial of Org 10172 in Acute Stroke Treatment. Stroke.

[53] Lammie G. (2000). Pathology of small vessel stroke. Br. Med. Bull..

